# Learning in a contextually complex rural clinical placement

**DOI:** 10.1007/s10459-025-10443-6

**Published:** 2025-06-05

**Authors:** Nicola Parkin, Kim Pearce, Rebecca Stengewis, Claire Drummond

**Affiliations:** 1 Flinders University Department of Rural Health, Adelaide, South Australia; 2 Rural and Remote Health South Australia, College of Medicine and Public Health, Flinders University, Adelaide, South Australia; 3 Adelaide, South Australia

**Keywords:** Rural clinical placement, Student learning, Learning in context, Context complexity

## Abstract

Introducing students to real-world contexts through clinical placements can provide rich learning experiences. In health professional education, these placements primarily focus on the supervised development of clinical skills within discipline-specific contexts. However, numerous implicit contexts influence the learning event, which may not be explicitly addressed in the placement’s curriculum or teaching structure but are nonetheless pedagogically significant. This study examines the distinctive contexts of a student-led, interdisciplinary Aboriginal allied health service placement in rural South Australia, highlighting the contexts’ unique experiential characteristics. We used a bricolage of phenomenological engagement and abductive thinking to investigate students’ experiences within and across these learning contexts. Students’ accounts revealed that their experiences at the intersections of these contexts held pedagogical significance. This finding prompts critical questions about the design and delivery of educational placements to maximise the inherent learning potential of contexts. We discuss the role of context in clinical placements and explore how context and context complexity can be effectively configured to support student learning across various clinical placement settings and models. We propose that developing context awareness and perspective, and attending to context convergence, can enhance meaningful learning in complex environments.

## Introduction

Rural clinical placements are pivotal in healthcare education, providing essential training opportunities for students preparing to work in underserved and diverse communities. Understanding the impact of contextual factors on learning outcomes in these placements is crucial for optimising the educational experience for students aiming to practice in rural settings. Recent research highlights the value of rural placements in preparing students for healthcare roles in underserved areas. For example McGrail et al. (2020) found that medical graduates who completed extended rural placements were more likely to pursue rural careers, demonstrating the positive effect of immersive rural experiences on workforce distribution. Likewise Fatima et al. (2018) emphasised the importance of contextually relevant learning in rural placements, showing that exposure to varied patient populations and healthcare models fosters adaptability and cultural competence.

Importantly, the success of rural placements is closely tied to the complexities inherent in rural healthcare, such as geographic isolation, limited resources, and disparities in access (McGrail et al., 2023; Ray et al., 2018; Seaman et al., 2022). These factors shape healthcare delivery and impact students’ readiness for practice. To maximise educational outcomes, educators and institutions need to address these contextual challenges in placement design (Swanson et al., 2024). Recent studies advocate for tailored interventions and support mechanisms to meet the specific needs of rural placement students (Green et al., 2022; Murray & Craig, 2019; Ray et al., 2018). Furthermore, reflective practices and debriefing sessions can enhance students’ critical analysis of their experiences, helping them recognise contextual influences on patient care and devise strategies for overcoming rural practice challenges (Smith et al., 2018). However, while contexts are recognised as important ‘for’ learning in rural clinical placements, contexts do not necessarily feature as important learning in themselves. Furthermore, while the complexity of the rural placement has been shown to be both a barrier and an enabler to learning (Hepburn et al., 2024), the complexity of those factors as *contexts* for learning within a placement experience has not been explicitly explored in the health professions education literature. As such, the role of context, and especially, of context complexit, in the rural clinical placement, invites further study. This paper explores the influence of context on placements in a student-led, interdisciplinary Aboriginal allied health service placement in rural South Australia, and its role in preparing healthcare students for rural and underserved practice settings. 

‘Context’, in the educational sense, is conventionally understood to be the setting and circumstances in which learning events take place (Patocka et al., 2024). Bates and Ellaway (2016) liken context to “dark matter” in the universe—an unseen yet vital element anchoring understanding. We regard context as an orienting nexus that shapes experience (Scharff & Stone, 2022), recognising that through understanding, we delineate this “dark matter” into discrete contexts with unique relevance (Scharff & Stone, 2022; Svensson, 2021). Our aim is to highlight the pedagogical potential of context complexity in rural health placements, thereby enriching students’ learning experiences and readiness for rural healthcare practice.

## Background: the contextually complex placement

The Riverland Interdisciplinary Student-led Placement Program (RISPP) is a contextually complex health professions placement delivered through Flinders University’s Rural and Remote Health, University Department of Rural Health South Australia (UDRHSA), in collaboration with a rural Local Health Network (LHN). Funded since 2023 by the Australian Commonwealth’s Rural Health Multidisciplinary Training (RHMT) initiative, RISPP aims to enhance rural health workforce capacity by providing impactful training experiences that encourage students to consider returning to rural practice upon qualification.

RISPP operates as an interdisciplinary, student-led allied health clinic situated within an Aboriginal Health Wellbeing Centre. The Centre primarily provides access to services for Aboriginal individuals and families but is also open to non-Aboriginal people who present at the Centre. Establishing the clinic as student-led addresses a gap in access to allied health services for the community and adds new opportunities for student placements across undergraduate and master awards. Students are housed in university accommodation in the rural community for the duration of their placement. A dedicated cultural education component ensures that the clinic operates in a culturally safe manner while enhancing students’ understanding of local cultural knowledge and history. Currently, three allied health awards at the University (speech pathology, physiotherapy and occupational therapy) offer students the opportunity to undertake this unique placement integrating multiple learning contexts. Placements across these disciplines range from five to ten weeks, with commencement dates staggered across the calendar year, rather than to an aligned cohort scheduling. RISPP students, where possible, are allocated placements in pairs to support the placement experience.

In this paper, ‘context’ refers to an encompassing frame of reference for a learning experience from which meaning can be derived, acknowledging that the same experience may have multiple frames of reference. The five RISPP learning contexts are: 1) clinical skills; 2) interdisciplinary practice; 3) rural; 4) Aboriginal cultural; and 5) student-led service. While also being aspects of the RISPP, each of these contexts draws its meaning from broader professional discourses which the student, on graduation into practice, will themselves be part of, either directly or peripherally. 

The clinical skills context involves discipline-related learning within the clinical setting, with the placement experience providing authentic, hands-on learning for developing the knowledge, skills, attitudes, and values essential for the workplace. In this context, learning occurs primarily “on the job” and may also include preparation, orientation, and complementary learning programs - such as the cultural education component embedded in the RISPP model. Clinical learning is supplemented by education about the healthcare system, preventive care, and the roles of health professionals (Dornan et al., 2006). Because the RISPP initiative aligns with the rural generalist model which deals with ‘complex wholes’ (Lynch et al., 2023), it accommodates a broader range of practice compared to more specialised clinical learning approaches.

The interdisciplinary context within RISPP involves the three allied health disciplines delivering a student-led service supported by an interdisciplinary and hybrid (Rudland et al., 2025) supervision model. Interdisciplinarity involves engaging with knowledge across disciplines and fostering new perspectives (Arthars et al., 2024) where, alongside clinical learning, students develop essential professional (and interprofessional) skills such as teamwork, leadership, and communication (Briggs & Fronek, 2020), aimed at building interprofessional practice (IPP) competencies that align with accredited practice standards (Hosny et al., 2024).

RISPP operates within a rural region of a population of approximately 6,000 and exposes students to the challenges of serving communities impacted by distance and limited access to health services (Bourke et al., 2004). Students are supported to provide services that are responsive to the local conditions and needs of the rural community (Gumede et al., 2021; Mpofu et al., 2014).

RISPP is delivered within the Aboriginal cultural context, located within an Aboriginal Wellbeing Centre. Aboriginal people face unique socio-cultural challenges that influence health service delivery, often exacerbated by disparities in access to culturally appropriate primary health services (Stuhlmiller & Tolchard, 2015). Student placements in Aboriginal communities offer invaluable learning experiences, fostering cultural respect and understanding while challenging negative stereotypes (Thackrah & Thompson, 2019), and support students to gain confidence in working with diverse populations (Kalistratova et al., 2024). However, evidence suggests that students may lack confidence when engaging in rural health practice with Aboriginal communities due to fears of causing offense (Bennett et al., 2013).

The RISPP model pivots on a student-led clinical service context. Student-led clinics (SLCs) are an established work-integrated learning strategy across disciplines, including allied health, and have shown measurable client improvement (Prestes Vargas et al., 2024; Schutte et al., 2015; Walker et al., 2024). SLCs may operate in hospitals, primary health clinics, community settings, or be established by universities to offer placement opportunities (Collis et al., 2024). They typically integrate into existing clinical services, but may introduce new health services where none previously existed (Briggs & Fronek, 2020; Varela et al., 2024).

## Methodology: a bricolage of phenomenological engagement and abductive thinking

This research draws on evaluation data generated by the RISPP students. In 2023, consultation-style interviews were conducted with students as they completed their placements, following protocols approved by Flinders University’s Human Research Ethics Committee (ID 5936). This ensured informed consent, safe data management, and student anonymity. Of the 12 students undertaking a RISPP placement, 11 consented to interviews, collectively representing experiences from the program’s inception (Varela et al., 2024). Of the 11 allied health students interviewed, three were enrolled in physiotherapy, three in speech pathology and five from the occupation therapy discipline. Following informed verbal consent, the semi-structured interviews lasted approximately one hour. These were primarily face-to-face (one was conducted online) and were audio-recorded. Students shared their experiences and opinions on the placement, together with any suggestions for placement improvement.

### Methodological approach

All four researchers on this study are UDRH staff interested in health workforce training and clinical practice models. NP is a rural health lecturer and the RISPP evaluator; KP is the UDRH Director and program lead; RS was also a rural health lecturer providing integrative student support; and CD, in a leadership role at RRHSA, contributed strategic input and experience in allied health education program development. The research team adopted a developmental and pluralistic approach from the outset, acknowledging and working with the diverse perspectives and philosophical views of its members. This collective stance purposefully fostered discussion about these aspects of conducting research and encouraged broadening our horizons through exposure to different modes of thinking. While some common ground could be identified, choosing a singular approach required compromise from the team. Consequently, we embraced an explicitly pluralistic methodological design—research bricolage—as our shared foundation.

Bricolage is a methodological approach emphasising flexibility, emergence, eclecticism, and plurality (Rogers, 2012) and is particularly suited for interdisciplinary research, capturing the rich interconnections and processes within phenomena (Kincheloe, [Bibr CR29][Bibr CR30]). The bricolage approach allows multiple methodological perspectives to coexist, which is arguably essential in a complex world, where a single orthodox methodology cannot adequately convey diverse meanings (Steinberg, 2015).

Our analytic approach combined phenomenological engagement *with* the data, and abductive thinking *about* the data. The two approaches are complementary. While abductive thinking is rooted in practice, phenomenology is grounded in a philosophical approach (Norlyk & Harder, 2010), yet both revolve on humanistic values (Shuster, 2012), and both *value what appears*. Phenomenology is concerned with the lived experience (Van Manen, 2014), while abductive thinking provides the framework for creative insight and sensemaking (Douven, 2021; Kolko, 2010; Schurz, 2008). By focusing on how phenomena show themselves and how they hide (Harman, 2007; Van Manen, 2007), phenomenology requires of the researcher immersive engagement rather than detached observation (Bortoft, 1996). Abductive reasoning, which operates between general and specific observations, reflects the ‘natural logic’ of qualitative research (Philipsen, 2018; Santaella, 2005), offering a practical and anticipatory analytic orientation (Chiffi et al., 2020).

### Analysis

The process of deriving meaning began with identifying and confirming the contexts (NP and CD). Subsequently, textual data from all transcripts was assigned to specific contexts (RS). Passages of text were de-identified, segmented into meaningful portions, and curated into a searchable document dedicated to each context, which was then analysed by the relevant research team member (NP, KP, or RS), according to their expertise.

Each researcher generated insights from their unique perspectives on the data. In keeping with both bricolage and abductive analysis, these insights were treated as provisional and tested for robustness in discussions with the entire research team, enabling multiple meanings to arise and synthesise (Kinn et al., 2013). The analysis progressed in three steps, moving from individual passages to broader contexts. The first step involved an immersive phenomenological engagement with the students’ actual experiences. Salient verbatim passages were identified and preserved to anchor meaning in the participants’ voices. In the next step, the research team noted emerging patterns of experience, and looked for connections, contrasts, and relationships between the contexts. Finally, we stepped back from the contexts themselves to surface insights ‘about’ contexts and their implications for health professions education.

## Results: experiences of learning in context

### Clinical skills context

Students had varied clinical experience prior to commencing the placement, with some students in their first year, and some in their final year of studies. Some students had previous and current experience working as allied health assistants. Physiotherapy students provided consultations for individuals referred to physiotherapy services; they also supervised and facilitated activities in the gym space. Speech pathology and occupational therapy students provided clients with six-week blocks of paediatric episodic care at the clinic. Students initiated the consultation at a ‘meet and greet’, followed by four-weeks of therapy and then a review consultation to ascertain if ongoing services were required.

Regardless of prior experience, all students were supported to develop *the full range of skills* needed to provide the clinic service to clients, including working with children under guardianship, working with carers, and with extended family members. Students also gained experience supporting warm referrals for clients to access additional health services.

The clinical process included demonstrating *accountability for the quality of their practices.* Critical thinking, collaboration, and reflective practice activities were scaffolded to support students to self-analyse their experiences, thoughts and actions, led by the lead clinical supervisor. Students reported on the *passion and enthusiasm of clinical educators*, which provided positive, person-centered role modelling and influenced future career choices.

### Interdisciplinary practice context

The students valued the learning that came from working in the interdisciplinary setting. Many of the interdisciplinary skills were introduced and supported by the lead supervisor as *transdisciplinary learning* skills, bringing to the fore those fundamental clinical skills common across the disciplines. However, especially initially, some role confusion did occur as students adjusted to working in this paradigm, while others reported missing out on some practical interdisciplinary opportunities due to program scheduling conflicts.

To support interdisciplinary practices, the students created a *collaborative physical space* in the Aboriginal Wellbeing Clinic. This enriched the experience of working together in the clinic, encouraging teamwork, communication, and creating a supportive working environment. In particular, the ‘meet and greet’ sessions were a good opportunity to undertake multidisciplinary screening and to practise *referring between the disciplines* as needed, within a ‘one stop shop’ approach.

RISPP students were guided through observation and feedback by *multiple supervisors*. The primary supervisor provided interdisciplinary supervision to all three disciplines; initially, a senior OT clinician, then a senior physiotherapy clinician. Students were also supported by discipline-specific clinical educators employed through the regional hospital network, thus making use of a ‘hybrid’ supervision model. Students received feedback from all of their supervisors, however the primary supervisor undertook the skills assessments required by the University.

The *interprofessional team* within the Aboriginal Health Wellbeing Centre provided a supportive environment where students developed practice-ready skills working with others across all aspects of service delivery, as a team. The cultural educators supported the students to reflect on ways that cultural learning could be implemented into clinical practice. The Health Service lead and administrators were proactive in checking in with the students, and open lines of communication between the team resulted in improved efficiency within the clinic.

As well as providing services together, students *learned together*. Wherever possible, students undertook interprofessional practices such as case conferencing. Peer support and bonding was an important feature of the placement experience, with students *living and working together*. Students reported that it was important to engage with others while on a rural clinical placement to create a sense of belonging. Students were often living together in shared accommodation for the duration of their placement, and they provided each other with friendship and support.

### Rural context

The students reported noticing the differences between rural and urban settings, specifically regarding the reduced availability of health services, coupled with a much thinner workforce. Students could see the realities of long waitlists in country areas, and this awareness supported a sense of *meeting a genuine need* in the community.

Some students remarked on the wider range of clinical presentations, and there was a distinct appreciation that rural healthcare professionals managed a constantly fluctuating role. Professionals working in the rural context were perceived to have a *broad scope of practice*, covering different client needs across multiple sites and locations. For some students this provided interest; others felt overwhelmed by the scope.

### Aboriginal cultural context

Students spoke of *developing cultural confidence* over the duration of the placement, and how the experience had enhanced their understanding of cultural safety and practice. Supported by the cultural learning component of the program and by the Aboriginal Wellbeing Centre staff, students developed a *culturally appropriate skillset*. For instance, they recognised the importance of developing and implementing a *relational approach* with Aboriginal clients and families. Building trust and rapport into practice took time but were understood as necessary for delivering appropriate care.

Students reflected that time away from the clinic with the cultural team brought a deeper sense of learning. In particular, the cultural immersion experience ‘on country’ (on culturally significant Aboriginal land) allowed the students to ask questions not deemed suitable in the clinic space. Together with the cultural learning program, this provided a valuable *insight into the wider context* of Aboriginal health.

### Student-led service context

The first students on placement experienced the raw beginnings of the service where processes were not yet fully established. Some of the first students told us that the lack of initial structure was challenging, revolving primarily on not knowing what to expect, or what was expected of them. For others, the experience of *being there at the beginning* of the service was something to be proud of.

All students were immersed in clinical service delivery from their first day. Independent practice was scaffolded according to their level of experience. As students demonstrated competence, their *autonomy increased*. Importantly, students were also empowered to determine the level of autonomy they felt comfortable with. This was valued by the students as a form of leadership.

From the beginning, students were given the *authority to design*. For the clinical sessions with clients, students worked independently or in pairs to develop the therapeutic goals and formulate the activities. More experienced students were free to bring in ideas they had used successfully in practice before. Students were also given the agency to shape the clinical space they were to use together in the service, working in consultation with the health service staff. Additionally, students had the responsibility of creating associated resources, which met both coursework-related requirements for their placement, and the practical necessities of the service. Resources included take-home information sheets, report templates, inventories, and process instructions.

*Practice appropriateness* was key to the design brief. Understanding the situational realities of the families they were caring for informed the design of appropriate therapeutic routines. Students checked in with the Wellbeing Centre staff regarding available resources and administrative procedures. Staff were also able to communicate and represent community perspectives about the clinic, providing a critical feedback loop for evaluating practice appropriateness.

Students understood that successful service delivery was as much about supporting clients to attend the clinic as it was offering therapeutic activities. It was clear from their accounts that students took personal and shared responsibility for the quality of the clinic service and were genuinely *invested in the clinic’s success*. Even while students were completing their placements at different times, their accounts demonstrated that they saw themselves as members of the service delivery team, albeit one with a constantly changing profile. Clinical care interruptions due to ‘gaps’ between student placements represented a challenge to continuity of client care. Responding to this, clinical care was ‘relayed’ between students and supported through handover notes. This longitudinal view of the service meant that even when service delivery improvements were not immediately operational, students were satisfied that they were prospectively improving the clinic service for the community.

NOTE: For insights in the students’ own words, please refer to Table 1*: The contexts, patterns of experience and indicative participant quotes.*

**Table 1:** The contexts, patterns of experience and indicative participant quotes

Patterns of experience within each context	Indicative participant quote
**Clinical skills context**	
The full range of skills	*We saw some great outcomes for some children. One of our kids got a diagnosis as well which will then get them onto [the National Disability Insurance Scheme].* (S7, OT) *I look at the patients notes, understand what they’re coming in with, make a rough plan for what to do, give my supervisor a call, or have a chat to them. Then the patient would come in. I see the patient. I go to my supervisor. I’d be like I did this, this, this, this, this went well.* (S1, PHYS) *My idea of OT got a bit too murky in going into speech and so the lines got a bit blurred, and I got bit too stuck in sitting in a speech mindset, even though they do cross over, I feel like I’ve bled a bit too much into that and I couldn’t necessarily pull my scope out of it.* (S3, OT)
Accountability for the quality of their practices	*I guess just how to be a better person and a better clinician. A lot of it is just being a good person, listening to consumers, listening to their concerns and taking all of that on and then coming in with your clinical reasoning. Learning how to build that interrelation between you and the consumer and the carers.* (S6, OT)
Passion and enthusiasm of clinical educators	*They were very passionate, motivated us, they were fun. I want to do that now.* (S10, SP)
**Interdisciplinary practice context**	
Transdisciplinary learning	*All those transdisciplinary things made what the placement is.* (S7, OT) *It made me a better speech pathologist … how little things that I can do in my speech pathology sessions that I can support from an OT perspective.* (S4, SP) *We had an occupational therapist for our clinical educator. Which was really good for learning interprofessional skills and multidisciplinary skills.* (S10, SP)
Collaborative physical space	*It doesn’t feel like you’re there by yourself for nine weeks. I think having one shared space is very good.* (S1, PHYS)
Referrals between disciplines	*We would all go and assess the child to see if they required a referral for both or just for one. And then based off of that, we’d either work together if they required OT and speech, or they would take them separately.* (S2, OT) *You can ask consumers, do you have trouble with your – showering? And if they do, I’m comfortable enough to tell them, you know OTs can help with this by doing this, this and this. Would you be interested in me making you a referral?* (S1, PHYS)
Multiple supervisors	*[The discipline supervisor] knows the speech pathology side of things, and [the lead supervisor] was more like those professionalism and communication and those kinds of attributes that we need to have. It was interesting having two people managing that assessment, but they did come together and work it all out.* (S4, SP)
Interprofessional team	*I worked really closely with one of the Aboriginal health workers, who worked with the bookings. She loved that spreadsheet idea that I did with the client list. She said it made such a big difference. I got really good feedback from her saying that I communicated well with her. But the spreadsheet was implemented in the sense that she could make changes that fit her and that would work with me. She loved it.* (S8, PHYS) *In the cultural learnings [the educator] would ask us ‘have you been implementing anything in the therapy?’ and stuff like that. And we were able to debrief that way.* (S2, OT) *That’s what made it the best placement, is just having all those people that you felt comfortable and supported with, and it just made life a lot easier.* (S8, PHYS)
Learning together	*All the students sit together, talk to their cases and get input.* (S1, PHYS) *We all bonded really well. And we have been able to bring that bond, you know, through games night. And, you know, like going out and stuff.* (S5, PHYS). *It was good to have that peer support and we’ve both got different skill sets and to brainstorm and work together. I do feel like the co-student peer support system kind of works well, rather than just being sort of isolated. At least you’ve got other students around that you can all develop your clinical knowledge together.* (S3, OT)
Living & working together	*What helped is that we all really got along together, we had a good friendship between the five of us.* (S7, OT) *Just when we were just sitting in the living room, they would be talking about a client that they were seeing or something they were studying for the next client and I pitch in a little bit like, “What’s that?” And then they’d teach it to me a little bit because I didn’t have a whole lot of knowledge about speech paths before. It was interesting just to hear their learning side of it.* (S8, PHYS)
**Rural context**	
Meeting a genuine need	*Just like seeing that pressure was – yeah, it was crazy.* (S10, SP) *You do see the sadder side of things too, because there are so many people that get put on the waitlist or are low priority.* (S11, SP) *The staff there were so great and passionate about the clinic and were just so grateful to have us there.* (S11, SP)
A broad scope of practice	*I was going from aged care in the morning to then seeing paediatric clients in the afternoon.* (S4, SP) *You’re not stuck in one location*. (S8, PHYS)
**Aboriginal cultural context**	
Developing cultural confidence	*I was very nervous the first couple of sessions … I did not want to be another one [barrier].* (S8, PHYS) *You can visualise that change [in knowledge] from the start to the end [of the placement].* (S8, PHYS)
A culturally appropriate practice skillset	*Like not having too many people in the room that aren’t Aboriginal because you don’t want that power imbalance*. (S6, OT)
A relational approach	*Everyone has different connection points.* (S5, PHYS).
Insight into the wider context	*[I got] perspective into Aboriginal people’s upbringing, and how it’s so different to mine.* (S9, OT) *Understanding the trauma and the trauma that’s passed down into generations really opened my mindset.* (S9, OT) *To understand why there are a lot of different health outcomes between Aboriginal people’s populations compared to white Australians.* (S9, OT)
**Student-led service context**	
Being there at the beginning	*It was all just so new and we were kind of working it out.* (S4, SP) *I wanted that reassurance and to talk through things.* (S4, SP) *It would definitely be something that I potentially put on my CV or bring up in an interview that I experienced.* (S3, OT).
Increasing autonomy	*We were just put into it … so we’d learn on the go.* (S9, OT) *At the start, [the clinical educator] walked me through it. So, when I was setting up the outcome measures, she gave me that idea and said a couple of ways that I could go about it, but I can pick. And then by the end, she was like, “I want you to identify yourself and do it yourself.” So, it was gradual. By the end of the placement, I was picking up things as I went.* (S8, PHYS) *[The clinical educator] was really good in that she was like, if you want me, I can be there. If you don’t, kick me out, that’s fine. Like, she let us lead that too, so we had leadership in that way. So pretty much, as much or as little as we wanted, which was quite nice.* (S11, SP)
Authority to design	*Making that a really fun, comfortable, nice space.* (S11, SP) *It was up to us how we would go about that.* (S2, OT) *I did implement some stretching plan, so I was able to make a few changes to that and everyone has been loving the stretch plan. I was also able to bring in more evidence-based management.* (S5, PHYS) *To make something that they need for the clinic.* (S9, OT)
Practice appropriateness	*If they didn’t show up to a session, we’d keep in contact with them to try to see what was going on.* (S2, OT) *Things I didn’t consider before and that made a world of difference.* (S8, PHYS) *A lot of the kids weren’t attending childcare and kindy. and it’s really important for them to be playing with children. When I asked parents or the caregivers if they would be interested in accessing the play group and stuff, they said yes. I went and sourced like a list of play groups and whatever else was in the area and gave them to the parents.* (S2, OT)
Invested in the clinic’s success	*They [the community] were raving about [the peanut ball] and how good it was. Just to get the community talking about one thing you’re using in session was huge. It was something so simple.* (S7, OT) *We’ve been really careful with planning [the service] as well. Otherwise, we might be setting up for failure.* (S5, PHYS) *It would be a good idea to have a visual: these are the [students] I’ve seen, this is the one that I did this with.* (S8, PHYS) *I could tell that [the supervisors] wanted to implement the strategies.* (S1, PHYS)

### Trans-contextual experiences

There were two kinds of experiences reported by students that were remarkable for their *trans*-contextual character: an expanded frame of care, and personal growth and transformation.

#### An expanded frame of care

Students discovered that their placement was not ‘typical’, and not just about providing a clinical service. As one student explained, this was “because of the nature of the clinic” (S5, PHYS), which compelled students to expand their frame of care beyond the strictly biomedical clinical paradigm they were used to.

Supported through the cultural learning program, students understood that the quality of their clinical care depended on more than transacting a service but was about establishing and sustaining genuine connections with the people they were caring for. The playgroup idea, explained one student, was not “a clinical thing” (S2, OT), but about meeting the needs of the community, grounded in the importance of connection within the rural context.

Students were taking all of the contexts into account and ‘seeing’ the possibilities of their practice from new perspectives, such as that of the community’s – as this student’s thinking shows:

You could have like a bonding [activity], like small games … or some fun activities where they can bring their families in. Then that shows the strength of the community. And that deepens their relationship with the student clinic as well. (S5, PHYS)

#### Personal growth and transformation

We heard from many of the students’ stories of personal growth and transformation - “lots of actual feel-good moments in that in that placement” (S11, SP). Some of the ‘feel-good’ value of the placement may have been associated to the novelty of the contexts for the students: all students experienced something new, and most of them had many new experiences. The many ‘firsts’ for students occurred across all of the contexts: first time living away from home and being away from their support networks of families and friends; first time spending time in a small country town; first time working with Aboriginal people; first time working with children; first time working in a multi-disciplinary setting; and first time working with many of the systems and practice demands expected of running a student-led service. These firsts were both exciting, and overwhelming at times.

While challenging, students also recognised the potential for growth inherent in these experiences: “I just thought it’d be, see patients, that’s it, but it was - you had to actually think a lot wider and a lot deeper than I expected” (S8, PHYS). The impacts of these new ways of thinking in new settings, with new people, was both personal and professional: “I definitely grew as a clinician, but also as a person as well” (S10, SP).

While students’ developmental experiences were guided to a degree, for the most part they were experience-led, with learning occurring through debriefs and reflective activities after the fact. The involved manner of their engagement brought their learning into view, as this student expressed with such clarity: “for me the way I like to learn is just being thrown into it” (S7, OT). Through immersion, students learned that they could “find my feet strongly [and] set boundaries as well” (S10, SP), and to “learn some skills on my own” (S9, OT). The expectations created some pressure, but “pressure makes diamonds, for me anyway … it was so much growth” (S10, SP).

It was clear from the students’ accounts that they rose to their responsibilities in part because it was necessary that they did so. As this student observed, if the lead supervisor had been present all the time, “we wouldn’t have learnt as much - when you just have to do it, you develop that confidence” (S7, OT).

Lastly, the following powerful account accents the opportunity that students had – and seized – to develop in ways they had not previously imagined:

[This has been my best placement, having] the time and space that I had to work on things, to think things through, to create things was just amazing. I’ve never had that much time in a placement just to do non-client things so that was great. I think some other placements are too focused on seeing as many people as you can, whereas this one was very focused on the learning side of it, the developing and everything like that. (S8, PHYS)

## Discussion: the role of context

Despite the modest number of students interviewed, their accounts clearly revealed the influence of contexts on their learning. Treated separately, each of the five pre-identified RISPP contexts supported differentiated learning outcomes and potential. We observed that regardless of their discipline or whether studying at postgraduate or undergraduate level, students encountered the full complexity of contexts during their placements. The combination of these contexts contributed significantly to the overall experience of the placement.

Students’ accounts highlighted that their experiences at the intersections of contexts had a unique pedagogical value – for instance, developing a culturally appropriate skillset is conditioned by both the Aboriginal cultural context and the clinical skills context. However, we noted two learning outcomes that appeared to arise at the cross-play of *all* the contexts. The first, the development of an expanded frame of care, showed a shift from a therapeutic focus to a relational one, moving from discipline to service, and from client to community. This exemplified a developing awareness of the ‘whole,’ encompassing systems, individuals, and experiences. Here, we can identify the beginnings of a naturally emerging ‘generalist’ ethos that embraces a relational and integrated approach to care (Lynch et al., 2023; Stange, 2009) sensitive to the inherent relationality of rural communities (Longenecker et al., 2025). This is in no small part because “there are impacts on service uptake and demand when people are known to each other” (Farmer et al., 2012 p. 1207), and represents a ‘meta’ form of caring that is considerate about how care is delivered. As Koens et al. (2005) assert, the commitment and meaning derived from learning in authentic practice contexts tend to be profound.

The second example of learning emerging at the intersection of the contexts concerned personal growth and transformation. Here, the student’s subjective experience itself became the context for learning. It is common for students on rural clinical placements to report highly personal experiences, such as developing self-worth (Bradley et al., 2020; Longman et al., 2020), enjoying autonomy (Glenister et al., 2024), and fostering positive relationships (Moran et al., 2024). Personal impacts such as these have the potential to carry through into practice (Swanson et al., 2024).

## Context complexity

Contexts are inherently complex; they do not exist as isolated entities but are intertwined and layered. Reflecting on our analysis, questions arise regarding context complexity: Does learning within one context gain depth from its proximity to others? Does the specific arrangement of contexts matter? Are there contextual tensions or synergies that particularly enhance learning? Does the award level make a difference to students’ awareness of the contexts they are learning within?

Although our initial analysis yielded many avenues for further research, our primary objective was to surface for investigation the awareness of context complexity in learning. The task itself is not straightforward. When addressing complexity, conceptual frameworks like the Cynefin model (Kurtz & Snowden, 2003; Snowden & Boone, 2007) can be invaluable. ‘Cynefin’ is a Welsh term that denotes the various factors in our environment and experiences that influence us in ways we may not fully comprehend, such as non-linear relationships, emergence, evolution, and unpredictability (Snowden & Boone, 2007). This speaks to Bates and Ellaway’s (2016) description of a context as a dynamic system arising from underlying patterns of people, places, and practices.

Embracing complexity necessitates an inside-out approach to learning, focusing on the experiential emergence of patterns, learning, and meaning as they develop. Pedagogically, contexts serve to make abstract content more tangible, enabling students to grasp the subject matter and apply it in various situations. As learners gain expertise, their practices become less abstract and more context-dependent (Burke, 2020), aligning with the goal of fostering ‘thinking in practice’ for our graduates (Forneris & Peden-McAlpine, 2006).

In practice placements, context is both total and experiential. Even within a purposefully contextual learning environment (Strasser & Neusy, 2010), context often serves content – though it can be a highly instructive servant. When made explicit, Bates and Ellaway’s (Bates & Ellaway, 2016) ‘dark matter’ (of context) can be transformed into ‘subject matter,’ allowing context to become content. However, context represents a unique form of content, retaining in itself a certain unknowability, especially when multiple contexts are interwoven.

The aim is to promote contextually sensitive clinical practice, which serves as an indicator of expertise (Robinson et al., 2022). This presents a dual opportunity: to learn about contexts themselves, and to discern contexts within any given practice situation. Given that our understanding of contexts is always partial and imperfect (Ellaway, 2024), the challenge lies in recognising contexts as complex phenomena—non-linear, dynamic, emergent, and unpredictable. 

To foster context awareness, practices of noticing (Clement et al., 2022; Dall’Alba, 2020; Orrell, 2019) should be integrated into placement settings. Contexts provide vantage points for observation and interpretation. Contextual perspective work entails immersing oneself in a context and experiencing its distinctiveness. When engaging with a problem or situation within a particular context, we privilege that context as a lens or standpoint (Green et al., 2022) for understanding the world. Engagement with multiple contexts further enriches understanding by bringing diverse perspectives on a singular problem or situation, however, it is integrating those contextual perspectives that leads to a more thorough and nuanced understanding. 

As we develop context awareness and perspective, the distinctiveness of contexts begins to diminish, revealing something more essential. This phenomenon was evident in the RISPP examples of expanded frames of care and personal growth. In both cases, the separate meanings associated with individual contexts converged into a fuller, more meaningful understanding: in the first instance, as meaningful practice; in the second, as significant personal experiences. These findings, separately and together, underline the importance of attending to the holistic development of human beings (Couper et al., 2024) - both pedagogically and therapeutically.

We conceive this phenomenon as *context convergence.* One way to understand a convergence of this kind is through transdisciplinarity, a field applying complexity theory to emergent practices (Lawless et al., 2024). Transdisciplinary approaches transcend the challenges of interdisciplinarity—such as commonalities, borders, and translation—to embrace the ‘necessary togetherness’ of disciplines and knowledge systems, a principle already demonstrated in practice (Scharff & Stone, 2022).

## Implications for placement design

Regardless of the placement model or the degree of context complexity, opportunities exist to enhance learning experiences through attention to contexts. While more research is needed, we suggest that developing context awareness and perspective, and harnessing the synergistic effects of context convergence, are worthy starting points. Importantly, we can support placement educators and supervisors to orient themselves to the pedagogical potential of contexts, so that they feel confident to support students to orient likewise.

To foster context awareness, students should be encouraged to identify *for themselves* the contexts they practice in, and how they are relevant to their learning. As outlined by Yacek and Gary (2020), emphasising moments of ‘epiphany’ or insight as they arise highlights how educators can recognise and amplify transformative experiences and support students to reshape their perspectives and practices in meaningful ways. Such practices encourage contextual perspective work – and ultimately, context converge - as they open diverse standpoints to view within the one experience. Educating at the intersection of contexts may not be intuitive for educators, yet it is critical for high stakes learning (Divjak et al., 2024)

Learning design is only beginning to attend to contexts and their complexity, and more work is needed. As Griffiths and Inman (2017) enjoin, learners and designers of learning alike need to “consciously consider the governing principles for [complex] contexts in order to improve the depth, completeness and security of learning” (p. 232), proposing that the Cynefin framework (Snowden & Boone, 2007) provides an appropriate organiser for the work. The Cynefin framework includes some practical tools that may guide placement design for complex contexts, such as opening discussion; establishing delineating boundaries; fostering resonance; encouraging dissent and diversity; and managing the conditions for emergence. While Snowden and Boone initially established these strategies to support the leadership context, they are arguably equally applicable to the learning domain.

## Conclusion

In this study we examined the complexity of contexts within an interdisciplinary, student-led allied health clinic located in an Aboriginal Health Wellbeing Centre in rural South Australia. We observed that, while students’ experiences of learning could be seen to fall primarily into any one of the contexts, pedagogical value seemed to be strongest at the intersection of contexts. We noted in particular two trans-contextual experiences as being of significant value: developing an expanded frame of care; and personal growth and transformation.

We propose that context awareness and perspective work (orienting to contexts) and context convergence (the potential synergy offered by context complexity) are tangible conceptual starting points for the important work of developing the pedagogical potential of complex contexts within our educational programs. These concepts can help us to frame new questions about the role of context in the purposes, conditions, practices and possibilities in our programs. Theoretical organisers such as the Cynefin complexity framework and transdisciplinarity may provide practical design ideas for the implementation of learning programs.

While particularly applicable to rural clinical placements, the ideas raised in this study are relevant to practice placements in general – and indeed, to any contextually-complex educational program. In all cases, what is important when designing and delivering learning, is to pay attention to the role of contexts in the learning experience.

While we are enthusiastic about these ideas, we recognise that we have only begun to explore the pedagogical potential of contextually complex learning environments - and the methods needed for investigating them. Further research is essential to understand how contextual complexity may influence students’ career intentions and readiness in rural health settings. Additionally, investigating the supports that educators and supervisors require to effectively assist students to learn in contextually complex experiential environments presents an important avenue for future inquiry.

## Data Availability

Data is provided within the manuscript.
